# New records of the endemic Sicilian land snail species Marmorana (Murella) muralis (O. F. Müller, 1774) from the north of Tunisia (Pulmonata, Gastropoda)

**DOI:** 10.3897/zookeys.775.25740

**Published:** 2018-07-19

**Authors:** Issaad Kawther Ezzine, Najet Dimassi, Beat Pfarrer, Khaled Said, Eike Neubert

**Affiliations:** 1 Laboratoire de génétique, biodiversité et valorisation des bio-ressources, Institut Supérieur de Biotechnologie de Monastir, Avenue Taher Hadded (B.P 74) Monastir 5000, Tunisia; 2 Natural History Museum Bern, Bernastr. 15, CH-3005 Bern, Switzerland; 3 Institute of Ecology and Evolution, University of Bern, CH-3012 Bern, Switzerland

**Keywords:** 16S, COI, anatomy, Marmorana (Murella) muralis morphology, *Murella nicollei*, Sicily, Tunisia, 16S, COI, anatomie, Marmorana (Murella) muralis, morphologie, *Murella nicollei*, Sicile, Tunisie

## Abstract

Marmorana (Murella) muralis is known as an endemic species of Sicily Island, which is introduced in many European countries. Here, M. (M.) muralis is recorded from the north of Tunisia. In order to confirm the identification of samples collected from several localities, shell morphology, details of genital organs and two mitochondrial markers (COI and 16S) were investigated. The results of the molecular study, as well as the morphological and anatomical studies confirm the identification of all Tunisian samples as M. (M.) muralis. The analysis of mitochondrial markers shows a low divergence between Sicilian and Tunisian samples suggesting a recent introduction of M. (M.) muralis to the North of Tunisia. The comparison of morphological characters of M. (M.) muralis with shell characters of *Murella nicollei* described by Pallary (1926) confirms that the latter should be considered as synonym of M. (M.) muralis.

## Abstract (fr)

Marmorana (Murella) muralis est connue comme une espèce endémique de l’île de Sicile qui est actuellement introduite dans plusieurs pays européens. Dans la présente étude, nous enregistrons, la présence de M. (M.) muralis dans différentes localités du Nord de la Tunisie. Dans le but de valider l’authenticité des spécimens collectés de différentes localités, la morphologie de la coquille, l’anatomie de l’appareil génital ainsi que deux marqueurs mitochondriaux (COI et 16S) ont été analysés. Les résultats de l’étude moléculaire ainsi que ceux de l’étude morphologique et anatomique confirment qu’il s’agit bien de l’espèce Sicilienne M. (M.) muralis. L’analyse des deux marqueurs mitochondriaux montre une faible divergence entre les populations Sicilienne et Tunisienne suggérant ainsi une récente introduction de cette espèce en Tunisie. La comparaison des critères morphologiques de l’espèce M. (M.) muralis avec les critères de la coquille de l’espèce *Murella nicollei* décrite par Pallary (1926) confirme que cette dernière doit être considérée comme synonyme de l’espèce M. (M.) muralis.

## Introduction

Land snails compose a group of invertebrates which are characterized by low mobility and dispersal capacity. The evolution of morphological characters within land snail species is widely influenced by the environmental and ecological conditions. Marmorana (Murella) muralis is an endemic helicid species from Sicily Island, which is characterized by an extremely high variability of shell morphology as well as molecular characters (Fiorentino et al. 2013). It was demonstrated that paleogeographical factors and environmental changes affected the shell morphology of M. (M.) muralis in Sicily (Fiorentino et al. 2013). This species was introduced by humans to many other European areas such as Tuscany in Italy, Sardinia, the Baleares, Portugal and Bouches-du-Rhône in France. Tunisia is a quite well sampled area as evidenced by Letourneux and Bourguignat (1887), who documented land snails from a plethora of localities. Interestingly, they never recorded the presence of Marmorana (Murella) Pfeiffer, 1877 in the area. It was Pallary in 1926, who was the first to describe a *Murella*, *Murella nicollei*, from Tabarka in northwest Tunisia.

Recent sampling efforts by the senior author revealed the presence of a Marmorana (Murella) taxon at several localities in the north of Tunisia. The present study aims to 1) identify the samples collected from Tunisia based on morphological and molecular characters, 2) determine the possible origin of each Tunisian population known and 3) clarify the status of *Murella nicollei* Pallary, 1926.

## Materials and methods

Living specimens were collected by hand at several localities in Tunisia during two periods: spring 2014, and winter 2015/2016. Geographic coordinates were recorded using a GPS device. For subsequent molecular analyses, specimens were preserved and stored in 80% ethanol until dissection and DNA extraction.

### Morphological and anatomical studies

First assessments of the shell morphological characters were done by using simple magnifying glasses. Preserved animals were dissected under a LEICA M212 stereo microscope using thin tweezers. The genital organs of the specimens were removed from the body, and the outer morphology of the complete hermaphroditic genital organ (situs) and further morphological details were investigated. After that, shells, genital situs, and details of the genital organs were photographed with a LEICA DFC 425 camera combined with a LEICA M205 C stereo microscope. The multifocal images were processed by using Imagic IMS software (Imagic, Switzerland).

### Molecular study

Ten specimens of M. (M.) muralis collected from northern Tunisia were used in this study. We also included sequences of Italian M. (M.) muralis specimens (Fiorentino et al. 2008; Fiorentino et al. 2010; Fiorentino et al. 2013; Neiber and Hausdorf 2015), *M. serpentina* (Férussac, 1821) (Fiorentino et al. 2008, Fiorentino et al. 2010), and M. cf. globularis (Philippi, 1836) (Fiorentino et al. 2008) for comparison with our specimens. Almost all cytochrome c oxidase subunit I (COI) haplotypes published by Fiorentino et al. (2013) were included in the study to estimate the divergence between Tunisian and Italian populations. *Macularia sylvatica* (Draparnaud, 1801) and *Macularia niciensis* (Férussac, 1821) were selected as outgroup (Neiber and Hausdorf 2015). All specimens used are listed in Table 1. Sequenced specimens are housed in the voucher collection of the NMBE (Naturhistorisches Museum der Burgergemeinde Bern).

**Table 1.** Taxa examined in this study: species, localities, voucher, and GenBank accession numbers for COI, and 16S fragments.

Species	Voucher number	Localities	Latitude	Longitude	GenBank accession numbers	
					COI	16S
M. (M.) muralis	NMBE 551462	Manzel Abderrahmen, Bizerte, Tunisia	37.232494°, 9.868065°		MG780362	MG774439
M. (M.) muralis	NMBE 551463	Manzel Abderrahmen, Bizerte, Tunisia	37.232494°, 9.868065°		MG780363	MG774440
M. (M.) muralis	NMBE 551464	Manzel Abderrahmen, Bizerte, Tunisia	37.232494°, 9.868065°		MG780364	MG774441
M. (M.) muralis	NMBE 551454	Manzel Jemil, Bizerte, Tunisia	37.249964°, 9.914793°		MG780365	MG774442
M. (M.) muralis	NMBE 551460	Manzel Jemil, Bizerte, Tunisia	37.249964°, 9.914793°		MG780366	MG774443
M. (M.) muralis	NMBE 551461	Manzel Jemil, Bizerte, Tunisia	37.249964°, 9.914793°		MG780367	MG774444
M. (M.) muralis	NMBE 551465	Haouaria, Nabeul, Tunisia	37.052299°, 11.010219°		MG780368	MG774445
M. (M.) muralis	NMBE 551457	Kelibiya, Nabeul, Tunisia	36.838017°, 11.115843°		MG780369	MG774446
M. (M.) muralis	NMBE 551458	Kelibiya, Nabeul, Tunisia	36.838017°, 11.115843°		–	MG774447
M. (M.) muralis	NMBE 551459	Kelibiya, Nabeul, Tunisia	36.838017°, 11.115843°		MG780370	MG774448
M. (M.) muralis [Fiorentino et al. 2010]	FGC 35940	Joppolo, Italy	–	–	EU189905	EU189872
M. (M.) muralis [Fiorentino et al. 2010]	FGC 35948	Marsala, Sicily, Italy	–	–	EU189904	EU189871
M. (M.) muralis [Fiorentino et al. 2010]	FGC 35922	Selinunte, Italy	–	–	EU189907	EU189874
M. (M.) muralis [Fiorentino et al. 2010]	FGC 36598	Fiumedinisi, Sicily, Italy	–	–	GU391370	GU391399
M. (M.) muralis [Neiber and Hausdorf 2015]	MN 503	Lazio, Italy	41.885278°, 12.480833°		KR705023	KR704983
M. cf. globularis [Fiorentino et al.2008]	FGC 35918	Caltabellotta, Sicily, Italy	–	–	EU189919	EU189886
M. (M.) muralis [Fiorentino et al. 2013]	H1	Erice, Sicily, Italy	–	–	JX827102	–
	H2	Erice, Sicily, Italy	–	–	JX827103	–
	H3	Erice, Sicily, Italy	–	–	JX827104	–
	H4	Erice, Sicily, Italy	–	–	JX827105	–
	H5	Erice, Sicily, Italy	–	–	JX827106	–
	H6	Monte Cofano, Sicily, Italy	–	–	JX827107	–
	H8	Erice, Sicily, Italy	–	–	JX827108	–
	H9	Monte Monaco, |Sicily, Italy	–	–	JX827109	–
	H10	Erice, Sicily, Italy	–	–	JX827110	–
M. (M.) muralis [Fiorentino et al. 2013]	H11	Monte Monaco, Sicily, Italy	–	–	JX827111	–
	H12	Erice, Sicily, Italy	–	–	JX827112	–
	H13	Erice, Sicily, Italy	–	–	JX827113	–
	H14	Monte Monaco, Sicily, Italy	–	–	JX827114	–
	H15	Monte Cofano, Sicily, Italy	–	–	JX827115	
	H16	Monte Monaco, Sicily, Italy	–	–	JX827116	–
	H17	Monte Monaco, Sicily, Italy	–	–	JX827117	–
	H18	Monte Monaco, Sicily, Italy	–	–	JX827118	–
	H19	Monte Monaco, Sicily, Italy	–	–	JX827119	–
	H20	Monte Monaco, Sicily, Italy	–	–	JX827120	–
	H22	Monte Monaco, Sicily, Italy	–	–	JX827122	–
	H23	Monte Monaco, Sicily, Italy	–	–	JX827123	–
	H24	Monte Monaco, Sicily. Italy	–	–	JX827124	–
	H25	Monte Cofano, Sicily. Italy	–	–	JX827125	–
	H26	Monte Cofano, Sicily, Italy	–	–	JX827126	–
	H27	Monte Cofano, Sicily, Italy	–	–	JX827127	–
	H28	Monte Monaco, Sicily, Italy	–	–	JX827128	–
	H29	Monte Monaco, Sicily, Italy	–	–	JX827129	–
	H30	Monte Monaco, Sicily, Italy	–	–	JX827130	–
	H31	Monte Cofano, Sicily, Italy	–	–	JX827131	–
	H32	Monte Cofano, Sicily, Italy	–	–	JX827132	–
	H33	Monte Monaco, Sicily, Italy	–	–	JX827133	–
	H34	Monte Monaco, Sicily, Italy	–	–	JX827134	–
	H35	Monte Monaco, Sicily, Italy	–	–	JX827135	–
	H36	Monte Monaco, Sicily, Italy	–	–	JX827136	–
M. (M.) muralis [Fiorentino et al. 2013]	H37	Monte Monaco, Sicily, Italy	–	–	JX827137	–
	H38	Monte Monaco, Sicily, Italy	–	–	JX827138	–
	H39	Monte Monaco, Sicily, Italy	–	–	JX827139	–
	H40	Monte Monaco, Sicily, Italy	–	–	JX827140	–
	H41	Monte Sparagio, Sicily, Italy	–	–	JX827141	–
	H42	Monte Sparagio, Sicily, Italy	–	–	JX827142	–
	H43	Monte Sparagio, Sicily, Italy	–	–	JX827143	–
	H44	Monte Sparagio, Sicily, Italy	–	–	JX827144	–
	H45	Monte Sparagio, Sicily, Italy	–	–	JX827145	–
	H46	Erice, Sicily, Italy	–	–	JX827146	–
	H47	Erice, Sicily, Italy	–	–	JX827147	–
	H48	Monte Monaco, Sicily, Italy	–	–	JX827148	–
	H50	Erice, Sicily, Italy	–	–	JX827149	–
	H51	Erice, Sicily, Italy	–	–	JX827150	–
	H37	Monte Monaco, Sicily, Italy	–	–	JX827137	–
	H38	Monte Monaco, Sicily, Italy	–	–	JX827138	–
	H39	Monte Monaco, Sicily, Italy	–	–	JX827139	–
	H40	Monte Monaco, Sicily, Italy	–	–	JX827140	–
	H41	Monte Sparagio, Sicily, Italy	–	–	JX827141	–
	H42	Monte Sparagio, Sicily, Italy	–	–	JX827142	–
	H43	Monte Sparagio, Sicily, Italy	–	–	JX827143	–
	H44	Monte Sparagio, Sicily, Italy	–	–	JX827144	–
	H45	Monte Sparagio, Sicily, Italy	–	–	JX827145	–
	H46	Erice, Sicily, Italy	–	–	JX827146	–
	H47	Erice, Sicily, Italy	–	–	JX827147	–
	H48	Monte Monaco, Sicily, Italy	–	–	JX827148	–
	H50	Erice, Sicily, Italy	–	–	JX827149	–
M. (M.) muralis [Fiorentino et al. 2013]	H51	Erice, Sicily, Italy	–	–	JX827150	–
	H52	Monte Monaco, Sicily, Italy	–	–	JX827151	–
	H53	Monte Monaco, Sicily, Italy	–	–	JX827152	–
	H54	Monte Monaco, Sicily, Italy	–	–	JX827153	–
	H55	Monte Monaco, Sicily, Italy	–	–	JX827154	
*M. serpentina* [Fiorentino et al. 2008]	FGC 35931	Siena, Italy	–	–	EU189932	EU189899
*M. serpentina* [Fiorentino et al. 2010]	FGC 32381	Sardinia: Casa Cantoniera, Italy	–	–	GU391369	GU391397
*Macularia sylvatica* [Neiber and Hausdorf 2015]	UB-ZMH-DNA-2843	Schaffhausen Switzerland	47.676389°, 8.614722°		KR705039	KR705002
*Macularia niciensis* [Neiber and Hausdorf 2015]	MN 2370-Hel-218	Provence-Alpes-Côte d’Azur_France	43.700000°, 7.241667°		KR705037	KR705000

### DNA extraction, PCR amplification and sequencing

Total genomic DNA was extracted from foot muscle tissue of each specimen using a standard phenol chloroform method (Estoup et al. 1996). Two mitochondrial gene fragments were chosen for analyses in the present study: cytochrome c oxidase subunit I (COI) of 711 base pairs (bp) length and the gene of the 16S ribosomal RNA subunit (16S rRNA) for an approximately 470–480 bp fragment. Polymerase chain reactions (PCR) were performed in a reaction mixture containing 15 ng of DNA template, 1X reaction buffer (1.5 mM), 0.1 mM of each primer pair, 0.2 mM dNTPs, Taq polymerase (1.25 U) and adjusted till a total volume of 25 μl with DNAase free water/sterilized water (UNIMED) (H_2_O). PCR reactions were run under the following conditions: 3 min at 95 °C, followed by 35 cycles of 1 min at 95 °C, 1min at 40 °C and 1 min at 72 °C and finally, 5 min at 72 °C for COI. For 16S the amplification conditions were: 3 min at 95 °C, followed by 35 cycles of 1 min at 95 °C, 1 min at 50 °C and 1 min at 72 °C, and finally, 5 min at 72 °C. PCR products were sequenced using automated and standardised ABI 3730 XL sequencing run with a read length up to 1.100 bp (PHRED20 quality) and using the same primers as for the PCR (Table 2).

**Table 2.** List of primers used for PCR and sequencing.

Gene	Name	Sequences	Reference
COI	COIFCOIR	5'-ACTCAACGAATCATAAAGATATTGG-3'5'-TATACTTCAGGATGACCAAAAAATCA-3'	Folmer et al. 1994
16S	16Sar16Sbr	5'-CGCCTGTTTATCAAAAACAT-3'5'-CCGGTCTGAACTCTGATCAT-3'	Simon et al. 1994

### Sequence analyses

Forward and reverse sequences were assembled, checked for ambiguities and aligned using the default settings of the ClustalW multiple alignment algorithm as implemented in Bioedit V 7.2.5 (Hall 1999) and trimmed for 655 bp and 414 bp respectively for COI and 16S. Obtained sequences were deposited in GenBank under the accession numbers MG780362-MG780370 and MG774439-MG774448 (Table 1).

Aligned Tunisian and Sicilian sequences were analysed using DnaSP v5.10.01 (Librado and Rozas 2009) to estimate the number of informative sites and nucleotide diversity for each marker. The K2P values were estimated using Mega v.6 (Tamura et al. 2013). The relationships of inferred haplotypes of Tunisian and Italian M. (M.) muralis were estimated using the Minimum Spanning Network (MSN) method (Bandelt et al. 1999) implemented PopART v1.7 (Leigh and Bryant 2015). Because of lack of sequences available on GenBank, we produced the haplotype network separately for COI and 16S markers.

### Phylogenetic analysis

Concatenated sequences of the two mitochondrial markers were analysed by Bayesian inference of phylogeny. The sequence data was initially partitioned into four partitions: three partitions corresponding to the codon positions of COI and one partition for16S. Based on the Akaike Information Criterion (AIC), the substitution models F81, K81uf+G, TrN+I and HKY+G were chosen as best models, respectively, for the first, second and third codon positions of COI and for 16S by PartitionFinder v 1.1.1 (Lanfear et al. 2012). For the Bayesian Inference, we used Mr Bayes v3.2.2 (Ronquist and Huelsenbeck 2003) using the partition scheme and substitutions models suggested by PartitionFinder. Four independent runs were conducted for 10^6^ generations, sampling every 1000. The first 25% trees were discarded as default burn-in and a majority-rule consensus tree was calculated from the remaining trees. Convergence between runs was assessed by comparing the traces using Tracer v1.5 (Rambaut and Drummond 2007). The topology obtained, and the posterior probabilities for each node were displayed with Figtree V1.4.0 (Rambaut 2012).

## Results morphology and anatomy

### Marmorana (Murella) muralis

Taxon classificationAnimaliaStylommatophoraHelicidae

(O. F. Müller, 1774)

#### Description.

**Figure 1.**
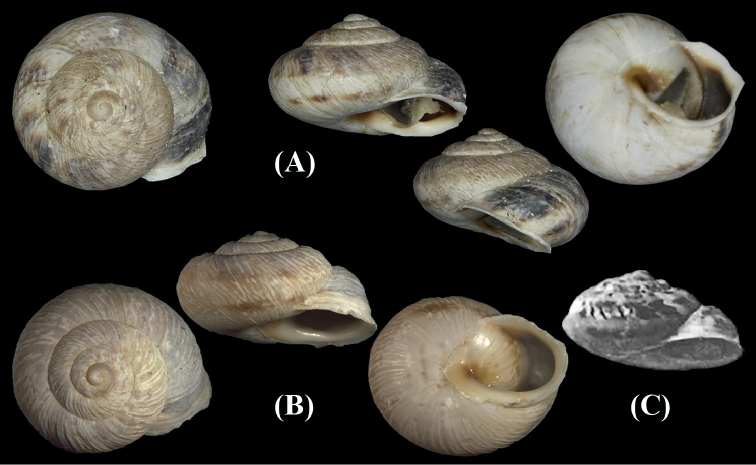
Marmorana (Murella) muralis (O. F. Müller, 1774) and *Murella nicollei* Pallary, 1926. **A** Menzel Jemil, Bizerte, 17.ii.2015, NMBE 534231, leg. Ezzine, D = 16.79 mm **B** Kelibiya, 10.i.2016, NMBE 551457, leg. Ezzine, D = 16.98 mm. Photographs Bochud & Ezzine **C** *Murella nicollei*, Tabarka. Scale bar: **D** 15.5 mm (copy of the original publication).

Shell medium-sized, depressed globular, thick, solid basic colour beige; large protoconch, clear, smooth, consisting of 1½ whorls; teleoconch consisting of 3½ slightly flattened whorls, distinctly ribbed; last whorl slightly keeled, larger than the rest whorls, descending towards aperture; aperture sub-spherical; peristome thick, white; suture moderately deep; underside with single interrupted spiral band; moderately ribbed, umbilicus completely covered by the reflected columellar margin (Fig. 1).

*Male genital anatomy*. Penis club-shaped, thick; epiphallus as long as penis; retractor muscle inserting into the distal part of the epiphallus; flagellum twice the length of epiphallus; penial papilla elongated, with a slit-like pore on one side.

**Figure 2.**
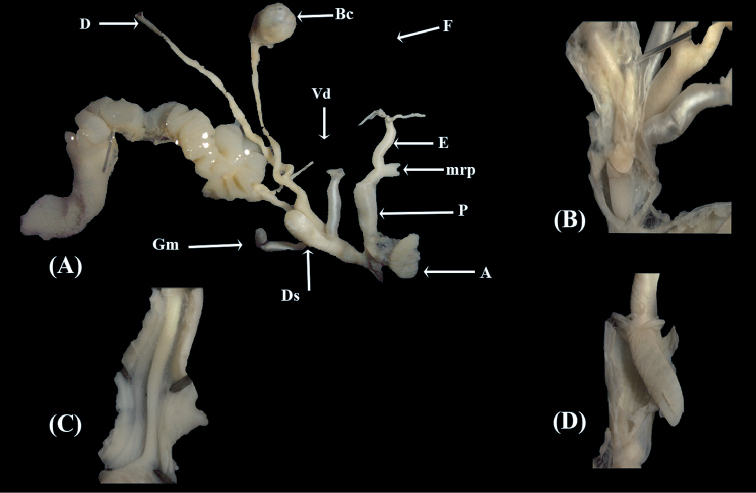
Anatomy of genital organs of Marmorana (Murella) muralis (Müller, 1774). **A** Situs **B** Details of dart sac **C** Details of epiphallus **D** Penial papilla. Abbreviations: A. atrium, Bc. Bursa copulatrix, D. Diverticulum, Ds. Dart sac, E. Epiphallus, F. Flagellum, Gm. Glandulae mucosae, mrp. Penial retractor muscle, P. Penis, Vd. Vas deferens.

*Female genital anatomy*. Dart sac simple, well developed, two glandulae mucosae, non-ramified, inserting into the middle part of the vagina near the base of the dart sac; bursa copulatrix and diverticulum inserting into the proximal part of the vagina (Fig. 2).

##### Haplotype network and genetic diversity

**Figure 3.**
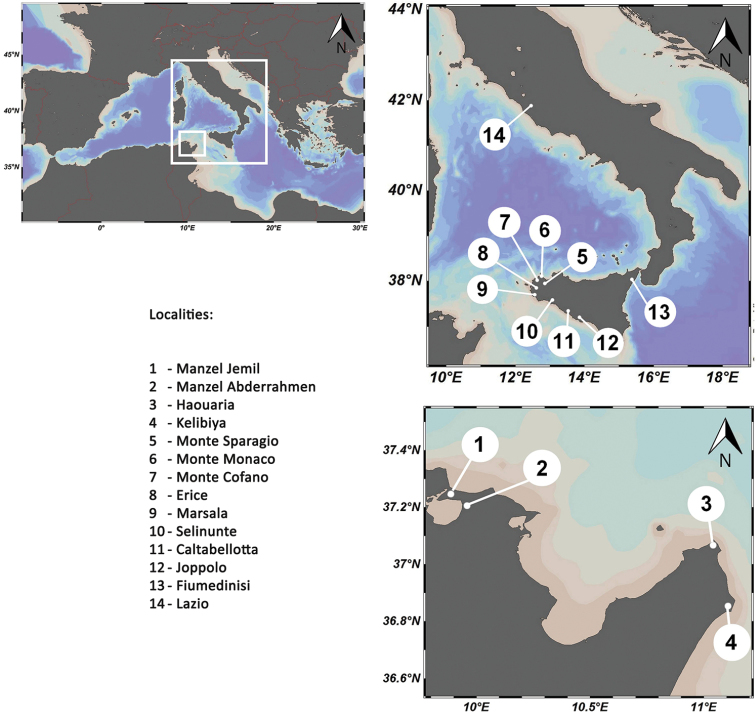
Localisation of Tunisian and Italian specimens used in the study.

**Figure 4.**
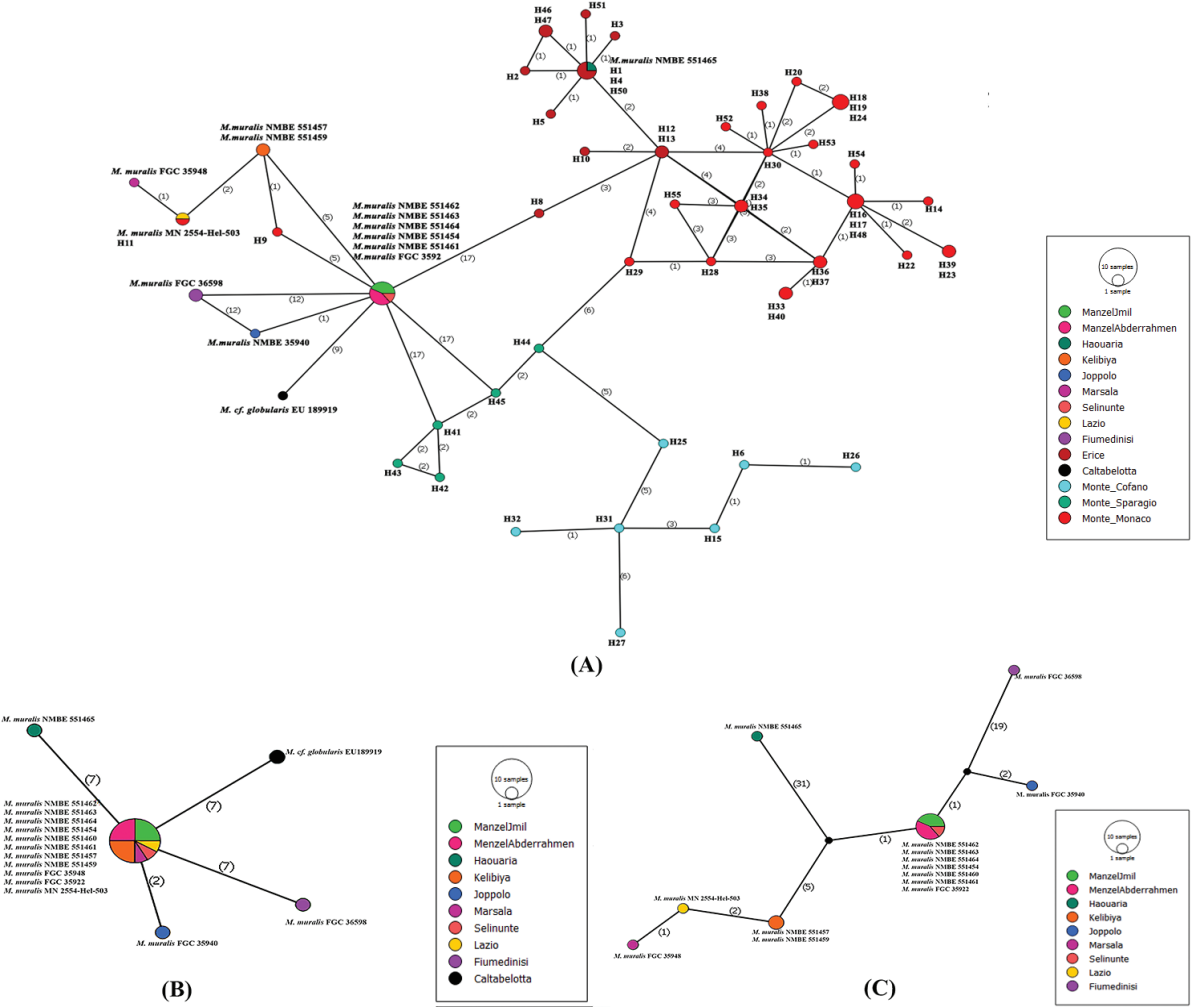
Haplotype network showing the relationships among Italian and Tunisian specimens of M. (M.) muralis. **A** Haplotype network based on partial COI sequences **B** Haplotype network based on partial 16S sequences **C** Haplotype network based on concatenated partial COI and 16S sequences.

Among nine Tunisian and 58 Italian partial COI sequences of M. (M.) muralis (Fig. 3), 46 distinct haplotypes were found, suggesting an extremely high haplotype diversity (Hd = 0.9815) (Fig. 4A). With 45 haplotypes detected, Italian sequences are highly diverse (Hd = 0.9903), while only 3 haplotypes were found in Tunisia (Hd = 0.5596). The haplotype network therefore suggests a relatively low genetic variability of COI sequences from Tunisian specimens compared to sequences from Italian specimens. Tunisian and Italian specimens share two haplotypes: the first is represented by the COI sequences of the samples collected from Manzel Jemil, Manzel Abderrahmen and the sequence from Selinunte. The second haplotype is represented by the sequence of the sample collected at Haouaria and the sequences H1, H4, and H50 from Erice (Fiorentino et al. 2013). The sequences of the samples collected at Kelibiya represent a unique haplotype that is neither shared with the other specimens from Tunisia nor with any of the specimens from Italy. Within the Tunisian sequences, the highest K2P value (0.078) was recorded between the haplotype of the sequence from Haouaria and the sequences from Kelibiya however; the lowest value (0.01) was reported between the sequences from Kelibiya and those from Manzel Jemil and Manzel Abderrahmen. Between Tunisian and Italian populations, the highest K2P value (0.081) was registered between the sequences from Kelibiya and the sequences from Erice (H5, H10) and Monte Monaco (H22, H28, H30, H38, H52). The lowest was recorded between the sequence from Haouaria and the sequences H1, H4 and H50 (Fiorentino et al. 2013) on the one hand and the sequences from Manzel Jemil- Manzel Abderrahmen and the sequences from Selinunte on the other hand (Fiorentino et al. 2010). The nucleotide divergence within Tunisian population reached a value of 0.0178 but the divergence was slightly higher (0.0316) within Italian population. Moreover, the divergence between Tunisian and Italian populations reached a value of 0.0353.

The analysis of ten Tunisian and six Italian 16S partial fragments shows five haplotypes suggesting a low haplotype diversity (0.450) (Fig. 4B). Sequences of Italian specimens represent four haplotypes (Hd = 0.80), while sequences from Tunisian specimens represent only two haplotypes (Hd = 0.20). Tunisian and Italian samples share one haplotype, which was represented by sequences of Tunisian specimens from Kelibiya, Manzel Jemil, Manzel Abderrahmen and sequences of Italian specimens from Lazio, Selinunte and Marsala. The sequences of specimens from Haouaria, Fiumedinisi, Caltabellotta and Joppolo represented four different haplotypes. The maximum value of K2P distance (0.02), within Tunisian sequences, is recorded between the sequence from Haouaria and the rest. While the maximum value recorded between Tunisian and Italian populations is 0.044 between the sequence from Haouaria and the sequences from Fiumedinisi and Caltabellotta. The nucleotide divergence of the 16S partial fragment is remarkably low within Tunisian population (0.00409), as well as, between Tunisian and Italian populations (0.00828).

The analysis of nine Tunisian and six Italian concatenated sequences (COI, 16S) recovered seven different haplotypes among them (Fig. 4C). One haplotype is shared by Tunisian and Italian populations. The rest is divided into two Tunisian and four Italian haplotypes.

##### Phylogeny

**Figure 5.**
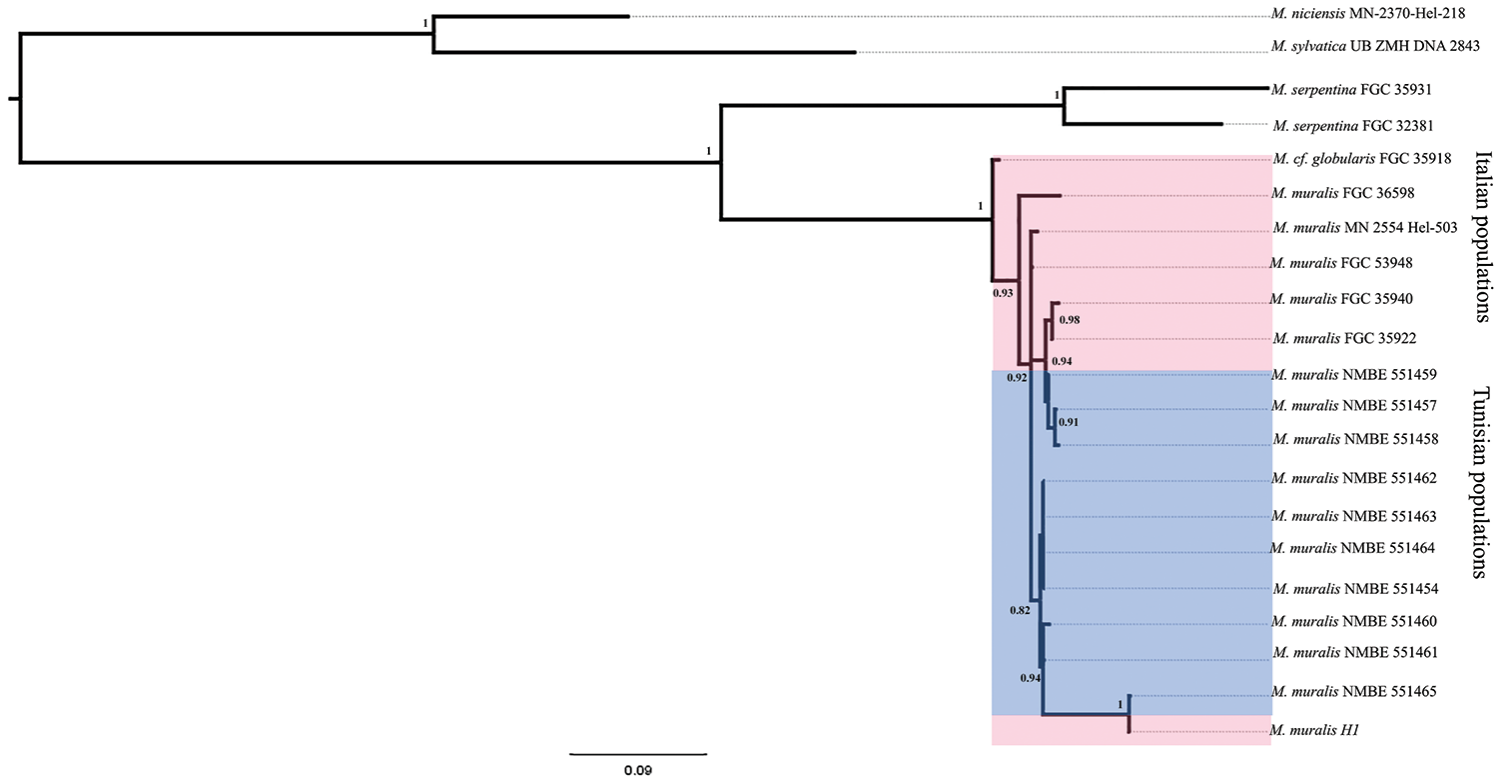
Bayesian 50% majority-rule consensus tree based on the analysis of concatenated partial COI and 16S sequences showing the relationships among Tunisian and Italian Marmorana (Murella) muralis samples.

The topology, obtained by Bayesian inference based on the concatenated COI and 16S data set was rooted with *Macularia sylvatica* and *Macularia niciensis* as outgroups (Fig. 5). The *Marmorana* species form two opposite clades well supported (PP = 1): The first one is formed by the samples of M. (M.) serpentina and the second is formed by both Tunisian and Italian M. (M.) muralis. Within the M. (M.) muralis clade, M. cf. globularis and the M. (M.) muralis of Fiumedinisi are situated at the base of the clade with a high value of posterior probability (1–0.93). The rest sequences form three well supported clades. The first is composed by the sequences of Marsala and Lazio, the second is formed by the sequences of Kelibiya, Joppolo, and Selinunte (0.92) and the third clade is formed by the sequences of Manzel Abderrahmen, Manzel Jemil, Haouaria and Erice (0.82).

## Discussion

### Morphology and anatomy

Marmorana (Murella) muralis is known as an endemic species of Sicily but it was introduced to several localities in southern Europe (Fiorentino et al. 2008). The morphological and anatomical characters of the Tunisian samples show the same morphological and genital anatomical traits presented by Fiorentino et al. (2010). Thus, these specimens are here considered to represent M. (M.) muralis. In Tunisia, this taxon was first recorded by Pallary (1926: 49, pl. VIII, fig. 9) under the name *Murella nicollei* from Tabarka. The photo of *Murella nicollei* (Fig. 1C) confirms the same shell morphological traits characterizing M. (M.) muralis. Thus, we consider *Murella nicollei* Pallary, 1926 to represent synonym of M. (M.) muralis. The species was probably introduced by Italian people, who lived in Tabarka for a long time. In fact, Italian people colonised Tabarka since the middle of the XVI century (Valérian 2012). The maximum number of Sicilian people settling in Tunisia was reached in 1891 (De Montety 1937). Since its description, there is no record of this species from Tabarka known to the authors. During the last decade, Tabarka was visited several times by Abbes and Ezzine, but neither empty shells nor living specimens of M. (M.) muralis could be found. The extinction of M. (M.) muralis in the area could be the result of 1) a negative ecological selection caused by the climatic conditions in Tabarka, or 2) the fragmentation and urbanisation of its habitat by human activities, which easily could reduce the population. Being an alien species to Tabarka it is quite possible that it could not well disperse in the area. As a result, the population is gone extinct. However, despite its extinction in Tabarka, it does well in the other Tunisian localities recorded here. Fiorentino et al. (2013) demonstrated that the shell morphology is highly affected by environmental changes in Sicily Island; the Tunisian populations seem not yet to be influenced by the new environment so far. The absence of any environmental effects on the shell supports the hypothesis that the species was quite recently introduced to the country.

### Network haplotype and genetic diversity

The nucleotide divergence of the COI sequences reaches a maximum value of 0.0316 (3.16%) between Tunisian and Italian populations. This value does not exceed the threshold of intraspecific divergence of land snails (4%) as suggested by Davison et al. (2009), and is comparable to the threshold of 3% suggested by Hebert et al. (2003) to characterize animal species in general. Furthermore, this value is smaller than the intraspecific divergence of the Tunisian *Xerocrassa latastei* reported by Ezzine et al. (2017). The comparison of the nucleotide divergence, the haplotype diversity, and the K2P value between Tunisian and Italian COI sequences shows a high diversity of this marker. The divergence between Tunisian and Italian populations might be the result of the isolation caused by the Mediterranean Sea, which can be considered a geographical barrier causing the restriction of passive gene flow between the two populations.

The analysis of the results obtained by the 16S sequences shows low values of nucleotide divergence, haplotype diversity, and K2P distance between Italian and Tunisian sequences, suggesting a weak diversity of this marker. The comparison of the parameters of the COI and 16S and the haplotype network show that COI is more polymorphic than 16S. COI seems to be suitable to estimate the divergence not only on species but also on population level. The haplotype network of the concatenated data confirms the results obtained by COI and 16S separately and shows that Italian populations are more diversified than the Tunisian ones. This supports the hypothesis of a recent introduction to Tunisia.

The haplotype network of COI sequences shows that the haplotype from Manzel Jemil and Manzel Abderrahmen is similar to the haplotypes from Selinunte and Joppolo, the haplotype from Haouaria is similar to the sequences from Erice, which can be interpreted as a hint to the origin of these particular populations. Interestingly, the haplotype from Kelibiya is unique and not shared with Italian populations. The divergence of the haplotype of Kelibiya may have two reasons: 1) these snails have been introduced from a genetically unknown population on Sicily, or 2) or it could be the result of the isolation of the population inside the castle. In fact, we visited Kelibiya several times, the population seems to be isolated but well adapted to the environment within the castle. The species does not live outside the castle. Geographical isolation is widely accepted to represent the main cause of genetic divergence within a species (Graybeal 1995; Baum and Shaw 1995; Olmstead and Reeves 1995). However, this is a process that requires many generations and might lead to changes in shell morphology as seen in Sicily. This is not the case here, so we assume that the first hypothesis has a higher probability.

### Phylogeny

The analysis of the topology obtained by the Bayesian Inference method shows that Tunisian specimens form one well supported clade (PP = 1) together with the Italian samples of M. (M.) muralis (Fig. 5) and thus proves that the Tunisian samples are conspecific with this species. The topology obtained did not divide the samples used into separate Sicilian and Tunisian clades, and the presence of a Tunisian or Sicilian ancestral clade could not be shown. Additionally, the shell morphology seems not to be affected by the environmental difference between Sicily and Tunisia, as might have been expected in case of a longer presence of the species in Tunisia.

To better understand the population dynamics of this species, more studies including more samples from Tunisia and from Italy will be needed.

## Conclusions

Based on morphological, anatomical, and mitochondrial markers, the present study confirms that the recently collected Tunisian samples of a *Marmorana* species belong to M. (M.) muralis. The absence of this species in the collection of Letourneux and Bourguignat (1887) leads to the hypothesis that the species may have recently been introduced to Tunisia, i.e. earliest after 1887. The first record for the species comes from Tabarka (Pallary 1926), but the species has gone extinct there. The recent populations from Tunisia share some Sicilian haplotypes indicating an origin from Selinunte, Erice and other well-known populations on Sicily; the population from Kelibiya is more isolated and does not relate to any genetically known population on Sicily. The haplotype networks of the COI, 16S and concatenated fragments prove that Italian populations are more diversified than the Tunisian. The shell morphology of the Tunisian populations is rather homogenous. We therefore conclude that the present distribution pattern is result of a recent anthropogenic introduction of the species in the north of Tunisia, which occurred sometime in the last 90 years. The species has to be considered a neozoon for the Tunisian malacofauna. It has to be emphasized that the development of the hitherto known four populations and their future dispersal in the country need to be observed. The impact of this alien species on the endemic land snail fauna of Tunisia needs serious future monitoring.

## Supplementary Material

XML Treatment for Marmorana (Murella) muralis
